# Rapid evolution of generalized resistance mechanisms can constrain the efficacy of phage–antibiotic treatments

**DOI:** 10.1111/eva.12653

**Published:** 2018-06-21

**Authors:** Claire E. Moulton‐Brown, Ville‐Petri Friman

**Affiliations:** ^1^ University of York York UK

**Keywords:** biofilms, coevolution, phage–antibiotic synergy, phages, *Pseudomonas aeruginosa*, resistance

## Abstract

Antimicrobial resistance has been estimated to be responsible for over 700,000 deaths per year; therefore, new antimicrobial therapies are urgently needed. One way to increase the efficiency of antibiotics is to use them in combination with bacteria‐specific parasitic viruses, phages, which have been shown to exert additive or synergistic effects in controlling bacteria. However, it is still unclear to what extent these combinatory effects are limited by rapid evolution of resistance, especially when the pathogen grows as biofilm on surfaces typical for many persistent and chronic infections. To study this, we used a microcosm system, where genetically isogenic populations of *Pseudomonas aeruginosa* PAO1 bacterial pathogen were exposed to a phage 14/1, gentamycin or a combination of them both in a spatially structured environment. We found that even though antibiotic and phage–antibiotic treatments were equally effective at controlling bacteria in the beginning of the experiment, combination treatment rapidly lost its efficacy in both planktonic and biofilm populations. In a mechanistic manner, this was due to rapid resistance evolution: While both antibiotic and phage selected for increased resistance on their own, phage selection correlated positively with increase in antibiotic resistance, while biofilm growth, which provided generalized resistance mechanism, was favoured most in the combination treatment. Only relatively small cost of resistance and weak evidence for coevolutionary dynamics were observed. Together, these results suggest that spatial heterogeneity can promote rapid evolution of generalized resistance mechanisms without corresponding increase in phage infectivity, which could potentially limit the effectiveness of phage–antibiotic treatments in the evolutionary timescale.

## 1 INTRODUCTION

Antimicrobial resistance and the emergence of multidrug‐resistant bacteria are global problems that are predicted to cause ten million deaths per year by 2050 (O'Neill, 2014). One alternative strategy to antibiotics is phage therapy: the use of parasitic viruses that specifically infect and kill certain bacterial pathogens. Compared to antibiotics, phage therapy is more targeted, leaving the beneficial or commensal microbiota unharmed (Skurnik, Pajunen, & Kiljunen, 2007), self‐replicating at the target site during the course of an infection (Carlton, 1999), efficient against antibiotic‐resistant bacteria (Kutter et al., 2010) and has low inherent toxicity to humans (Abedon & Thomas‐Abedon, 2010). Furthermore, phages have the distinct advantage of being able to evolve with the bacteria to regain infectiveness via a coevolutionary arms race (Betts, Vasse, Kaltz, & Hochberg, 2013; Friman et al., 2016; Scanlan, Buckling, & Hall, 2015).

Instead of replacing antibiotics, phage therapy could be used to complement antimicrobial therapeutic strategies (Torres‐Barceló & Hochberg, 2016). A growing body of evidence suggests that combinations of phages and antibiotics are more effective at controlling pathogenic bacteria than either treatment alone due to additive effects or phage–antibiotic synergy (PAS). Additive effects are expected when phage and antibiotic work independently of each other, and their combined effect is the sum of their independent effects. In the case of synergistic effects, phage–antibiotic combinations are often observed to have more detrimental (or less detrimental) effects on the pathogenic bacteria than would be expected based on the sum of their independent effects (Comeau, Tétart, Trojet, Prère, & Krisch, 2007; Hagens, Habel, & Blasi, 2006; Huff, Huff, Rath, Balog, & Donoghue, 2004). PAS is thought to occur because two sufficiently different selective pressures are more likely to kill both nonresistant (susceptible to antibiotics) and antibiotic‐resistant (susceptible to phages) pathogen genotypes (Torres‐Barceló & Hochberg, 2016). Moreover, two concurrently acting selection pressures might impose evolutionary trade‐offs for the pathogen, which could constrain the evolution of resistance to antibiotics, phage or both (Chan et al., 2016; Torres‐Barceló & Hochberg, 2016; Wang et al., 2017). Here, we studied this using a model system where we exposed *P. aeruginosa* pathogen to both phage and sublethal concentration of gentamycin and monitored ecological and evolutionary dynamics in both homogenous (planktonic) and spatial (biofilm) dimensions of the microcosms. In particular, we explored how phage–antibiotic combination affects the evolution of resistance mechanisms and how this process depends on the spatial structure of the environment typical for clinical infections.

The evolutionary responses to phage–antibiotic combinations could be explained mechanistically by population density effects, collateral sensitivity or by high costs of adaptation. First, if phage–antibiotic combination is able to reduce pathogen densities more clearly compared to single‐therapy treatments, it might limit the emergence of resistance mutations to either therapeutic agent via lowered mutation supply rate. Moreover, antibiotics and phages could be applied sequentially, which could limit the emergence of double‐resistant mutants if both antimicrobial agents are able to eliminate pathogen genotypes that are resistant to the other antimicrobial, that is, if phages can kill antibiotic‐resistant bacteria and vice versa. Second, it is possible that evolution of resistance to one antimicrobial agent leads to collateral sensitivity to another antimicrobial agent (Imamovic & Sommer, 2013). For example, a *Pseudomonas aeruginosa*‐specific phage has been reported to use outer membrane porin M (OprM) of the multidrug efflux systems MexAB and MexXY as a receptor‐binding site (Chan et al., 2016). Mutations that change the structure of these receptors has been shown to confer *P. aeruginosa* resistance to phages, but at the same time increase its susceptibility to antibiotics due to its less functional efflux pump (Chan et al., 2016). In the same way, it has recently been reported that phage selection can make *Ralstonia solanacearum* plant pathogenic bacterium more susceptible to antibiotics produced by *Bacillus amyloliquefaciens* biocontrol bacterium even though the mechanism has not yet been described in detail (Wang et al., 2017). Third, selection by phages and antibiotics could lead to increased costs of resistance, which could limit bacterial growth and infectiveness (Andersson & Hughes, 2010; Friman et al., 2016; Mumford & Friman, 2017). In support for this, it has been shown that phage selection can reduce *P. aeruginosa* and *R. solanacearum* growth and potential competitive ability most when bacteria evolved in the presence of both phage and antibiotics (Torres‐Barceló, Franzon, Vasse, & Hochberg, 2016; Wang et al., 2017), while phage resistance mutations have been shown to impair genes that are used for virulence leading to weaker infections (Addy, Askora, Kawasaki, Fujie, & Yamada, 2012; Chaturongakul & Ounjai, 2014; Torres‐Barceló et al., 2016).

Thus far, phage–antibiotic combinations have predominantly been studied in relatively homogenous laboratory environments. Within‐host environments are however often spatially heterogeneous (Costerton, Stewart, & Greenberg, 1999) allowing pathogens to grow as a biofilm—adhered to various surfaces and embedded in a protective matrix of extracellular polysaccharides, proteins and DNA. Biofilms are also prevalent and persistent in clinical settings and have been found to form on clinical equipment such as catheters (Trautner & Darouiche, 2010), intravenous lines (Percival, Suleman, & Donelli, 2015) and prosthetic heart valves (Donlan & Costerton, 2002). Biofilms typically have much higher antibiotic resistance than their planktonic counterparts (Stewart & Costerton, 2001), and there are multiple hypotheses for why this occurs. One explanation is that biofilm acts as a diffusion barrier preventing the penetration of antibiotics (de Beer, 1997). It has also been suggested that biofilms allow the persistence of pathogen subpopulations that can repopulate the biofilms when damaged by antibiotics (Cochran, McFeters, & Stewart, 2000). Third, the chemical microenvironment within the biofilm with zones of nutrient and oxygen depletion or waste accumulation could prevent the optimal functioning of antibiotics (de Beer, Stoodley, Roe, & Lewandowski, 1994). For example, some antibiotics target only actively growing bacteria and are thus ineffective against slowly reproducing bacteria within a nutrient‐ and oxygen‐depleted biofilm (Tuomanen, Cozens, Tosch, Zak, & Tomasz, 1986).

Biofilm formation has also been shown to be an effective general resistance mechanism against phages (Stewart & Costerton, 2001; Vidakovic, Singh, Hartmann, Nadell, & Drescher, 2018). Similar to antibiotics, extracellular polysaccharides and proteins could prevent phage adsorption to its receptors while metabolically less active cells are less likely to get infected (Abedon, 2017; Labrie, Samson, & Moineau, 2010). However, certain phages have the ability to produce enzymes, which can degrade the extracellular polymeric matrix (Hanlon, Denyer, Olliff, & Ibrahim, 2001; Hughes, Sutherland, Jones, & Rutherford, 1998), while other phages are able to propagate radially through a biofilm (Doolittle, Cooney, & Caldwell, 1996). Surprisingly, little is currently known about phage–antibiotic effects in biofilms. So far, it has been reported that treating *Klebsiella pneumoniae* infections with a combination of phage and antibiotic is more successful at limiting the emergence of phage‐resistant pathogen mutants compared to antibiotic alone, even though no difference was observed in the effectiveness to eradicate biofilms (Verma, Harjai, & Chhibber, 2009). Another study found that phage–antibiotic combinations were more efficient at eradicating the planktonic and biofilm populations of multiple *P. aeruginosa* strains compared to antibiotic‐alone treatments, but the evolution of resistance was not measured in this study (Nouraldin, Baddour, Harfoush, & Essa, 2016). Of late, it was shown that phage–antibiotic combinations can considerably vary in their efficiency to eradicate *P. aeruginosa* biofilms depending on the specific phage species and the class of antibiotic (Chaudhry et al., 2017). However, there are no studies addressing the efficiency of phage–antibiotic combinations on both planktonic and biofilm populations of pathogenic bacteria in long‐term evolutionary experiments. Moreover, there are no studies looking at phage coevolution during phage–antibiotic treatments.

Here, we used an experimental evolution approach to study the effects of phage–antibiotic combinations on *Pseudomonas aeruginosa* population density dynamics and resistance evolution. *Pseudomonas aeruginosa* is a Gram‐negative, opportunistic bacterial pathogen that readily forms biofilms. It is considered to be a leading source of nosocomial infections and is commonly linked with chronic diseases such as cystic fibrosis, where it is a major cause of morbidity and mortality (J. C. Davies, 2002). *Pseudomonas aeruginosa* infections are often difficult to treat because of low antibiotic susceptibility and the emergence of multidrug‐resistant strains (Lister, Wolter, & Hanson, 2009). We set up four treatments where we evolved *P. aeruginosa* in the presence of gentamycin, phage 14/1 or both for 15 days under highly controlled laboratory conditions (*N* = 5). In order to study changes in both planktonic and biofilm populations, all microcosms were seeded with 25 glass beads to allow destructive sampling and isolation of biofilm bacterial populations (one bead per treatment replicate at every sampling time point, i.e., every third day). In addition to measuring phage and bacterial population density dynamics, we quantified changes in pathogen resistance to antibiotics and phages, associated costs of resistance and phage–bacteria coevolutionary dynamics during the experiment.

## 2 MATERIALS AND METHODS

### 2.1 Media, strains and growth conditions

*Pseudomonas aeruginosa* PAO1 was used as a bacterial pathogen in this study. Isogenic starting stocks were cultured from a frozen ancestral stock in Lysogeny broth (LB) media and spread on LB agar plates. A single colony was chosen and used as a starting culture in the selection experiment. Phage 14/1 (Table 1) was used as a specific *P. aeruginosa* lytic phage. Phage stock solutions were prepared by growing frozen phages with PAO1 in LB media at 37°C for 24 hr with shaking at 200 rpm. To purify phages from bacteria, 10% (v/v) chloroform was added and cultures vortexed and centrifuged at 11,000× *g* for 5 min. Phage stocks were stored at 4°C.

**Table 1:** Strains used in this study^a^

Microbe	Strain	Source	Year	Morphotype	Serology group	Genus	Genome size (kb)
Bacteria	PAO1	Clinical, nonrespiratory, Australia	1954	‐	‐	Pseudomonas	6,264
Phage	14/1	Sewage water, Regensburg, Germany	2000	Myoviridae; A1	E serogroup	Pb1‐like virus	66

Note

a Source and genome size are given according to Friman et al. & Stover et al. (Friman et al., 2016; Stover et al., 2000); morphotype and serological grouping are given according to Merabishvili et al. (Merabishvili et al., 2007).

### 2.2 Experimental design of the selection experiment

Four evolutionary treatments were established with five replicate microcosms per treatment. Treatments included *P. aeruginosa* evolving alone (control), in the presence of gentamycin antibiotic, in the presence of phage 14/1 or evolving in the presence of both phage and antibiotic (combination treatment) (Supporting information Figure S1). We used 30 ml glass universals as microcosms that contained 6 ml of LB and 25 submerged glass beads (VWR). Previous research has shown that biofilms can form on the outside of plastic beads and can be successfully isolated using bath sonication (Traverse, Mayo‐Smith, Poltak, & Cooper, 2013). In this study, this method was modified with 4‐mm‐diameter glass beads as a scaffold for biofilms to form on. The bead biofilm model allowed us to destructively sample individual beads throughout the evolutionary experiment and study the effects of phage and antibiotic selection on biofilm populations separately.

All microcosms were initially inoculated with approximately 10^9^ cells/ml of isogenic stocks of PAO1. Phage treatments were further inoculated with approximately 10^4^ particles/ml of lytic phage 14/1. Antibiotic treatments contained 3 μg/ml of gentamycin in LB, which was tested and deemed as a sublethal concentration of the broad‐spectrum aminoglycoside antibiotic (Supporting information Figure S2). It was also found that gentamycin and phage 14/1 had additive effects over a 24‐hr growth period: Bacteria grown for 24 hr had lower densities at higher concentrations of gentamycin (antibiotic concentration: F_7, 48_ = 255.111, *p* < 0.001), and the addition of phage 14/1 additively reduced bacterial densities (phage: F_1, 48_ = 908.178, *p* < 0.001; phage × antibiotic concentration: F_7, 48_ = 19.222, *p* < 0.001; Supporting information Figure S2). In particular, the effect of phage was not significantly different at 0 and 3 μg/ml of gentamycin, which is indicative of additive effects instead of phage–antibiotic synergy (Supporting information Figure S2).

Microcosms were incubated statically at 37°C and maintained in fed‐batch culture throughout the experiment, where the volume of removed sample (83% of total volume) was replaced with fresh media (LB or LB with 3 μg/ml gentamycin), minimizing disruption to the biofilm. It was experimentally confirmed that 3 μg/ml gentamycin in LB remained effective at reducing bacterial density after 72 hr at 37°C (antibiotic: *t* = −27.1, *df* = 22, *p* < 0.001; Supporting information Figure S3). Therefore, microcosms were grown over 15 days, with sampling every 72 hr, which equals approximately 300 bacterial generations. At every sampling, a total of 5 ml of media was removed aseptically from each microcosm along with one randomly selected glass bead that was covered in biofilm (with sterilized forceps).

### 2.3 Isolation and population density measurement of planktonic and biofilm bacteria

Bacterial densities of planktonic and biofilm bacteria were measured throughout the experiment as colony‐forming units (CFU/ml). Planktonic bacterial population densities were measured by diluting and plating subsets of collected aquatic samples at every sampling time point. To measure changes in biofilm bacterial population densities, biofilm colonies were isolated from glass beads from each replicate population using a previously developed method (Popat et al., 2012). In brief, biofilm cells were collected by aseptically retrieving a glass bead from each tube, gently washing it three times in 5 ml phosphate buffered saline (PBS) using five inversions and transferring to 1 ml fresh PBS. The washed beads were then sonicated in a bath sonicator for 10 min and vortexed for 20 s. Recovered biofilm populations were diluted and plated on LB agar plates to measure colony‐forming units (CFU) per bead.

**Figure 1:**
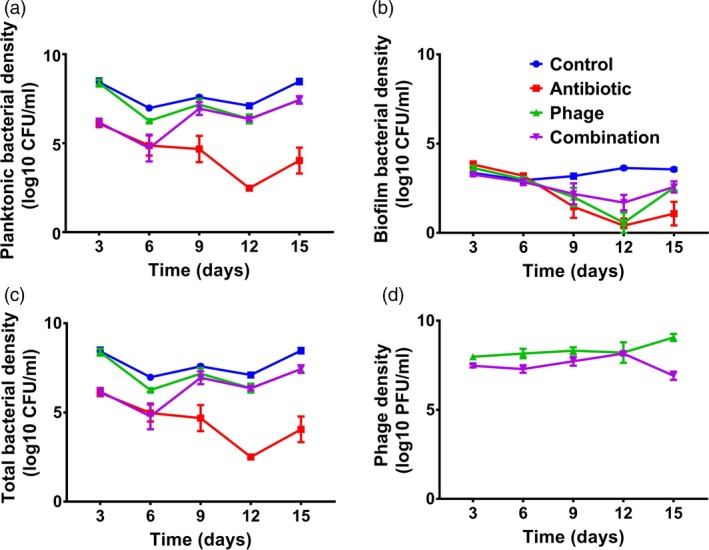
Bacterial and phage densities during the selection experiment. Panel (a) denotes planktonic bacterial densities (CFU/ml); panel (b) denotes total biofilm bacterial densities (CFU/ml; standardized with the total number of beads per microcosm); panel (c) denotes total bacterial densities (CFU/ml; sum of total planktonic and biofilm populations), and panel (d) denotes phage densities (PFU/ml). Different line colours refer to antibiotic‐only (red), phage‐only (green), combination (purple) and control (blue) treatments. Error bars show ±1 standard error of mean

To estimate total biofilm population densities per microcosm, CFU count obtained from one individual bead was multiplied with the number of total beads present in the microcosm at given time point (removed beads were not replaced with new beads during the experiment). Biofilm population densities were normalized similarly with each replicate population, and the Figure 1b shows the mean of total biofilm densities. Total bacterial population densities per microcosm were then determined as a sum of CFU of planktonic and biofilm populations per replicate population for every sampling time point. To measure changes in bacterial fitness and resistance over time (see description below), 12 colonies per replicate population were randomly isolated at every time point from both planktonic and biofilm population samples from each microcosm. All the isolated colonies were grown on 96‐well microplates overnight at 37°C in LB media and frozen at −80°C in 20% (v/v) glycerol.

### 2.4 Isolation and population density measurement of phages

To measure phage population densities and changes in phage infectivity and bacterial resistance over time, phage populations were isolated at every sampling point alongside bacteria. Phages were sampled from the liquid media and extracted using 10% (v/v) chloroform, vortexing and centrifugation at 11,000 × *g* for 5 min. The densities of phages retained in the supernatant were determined as plaque‐forming units (PFU/ml) using serial dilutions of phage suspension and the agar overlay method (Adams, 1959). In brief, cultures of phage‐sensitive ancestral PAO1 strain were grown for 24 hr at 37°C with shaking at 200 rpm, after 100 μl of PAO1 culture was added to 10 ml molten overlay agar and mixed by inversion. PAO1‐soft agar mixture was plated onto square Petri dishes on top of a layer of LB agar, and then, 5 μl of each phage dilution was pipetted onto the plate (totalling 48 dilutions per plate). Once dried, plates were incubated at 37°C for 24 hr and phage plaques were counted to ascertain phage concentration in PFU/ml.

### 2.5 Antibiotic resistance assay

Antibiotic resistance assays were conducted using the clones isolated in the end of the experiment. To this end, frozen plates of evolved biofilm and planktonic bacteria were fully thawed and replicated into 96‐well microplates with fresh LB using a pin replicator (~0.2 μl transfer per pin, Boekel). All evolved colonies were grown for 24 hr at 37°C after all bacterial isolates were replicated into fresh 96‐well plates containing LB with 3 μg/ml gentamycin. Bacterial isolates were further incubated for 24 hr at 37°C after which the growth was determined as optical density (OD_600 nm_, Tecan Infinite 200). Antibiotic resistance was determined as the bacterial growth in the presence of 3 μg/ml gentamycin relative to bacterial growth in the absence of gentamycin. Larger difference thus denotes for antibiotic resistance (growth data shown in Supporting information Figure S4).

### 2.6 Phage resistance assay

Phage resistance assays were conducted using the clones isolated in the end of the experiment. Evolved bacterial isolates were thawed and replicated into 96‐well plates containing LB media and inoculated with approximately 10^2^ particles of ancestral phage 14/1. After 24 hr of incubation at 37°C, phage resistance was measured in terms of optical density (OD_600 nm_) where higher growth of evolved bacteria in the presence of phage relative to density of bacteria grown without phage denotes the evolution of phage resistance (growth data shown in Supporting information Figure S4). Phage resistance was also measured every second time point throughout the experiment using streak assay (Buckling & Rainey, 2002) as a part of time‐shift assay (see below).

### 2.7 Time‐shift assay: detecting coevolutionary changes between bacteria and phage

A time‐shift assay approach (Buckling & Rainey, 2002; Nee, 1989) was used to determine whether bacteria and phages evolved adaptations and counteradaptations during the selection experiment. To this end, bacteria and phages were isolated from different sampling points during the experiment (transfers 1, 3 and 5). At every sampling point, the “contemporary” bacteria were challenged to phages isolated from two transfers in the “past” and the “future.” If bacteria and phages are coevolving to become more resistant and infective, respectively, “contemporary” bacteria should be more resistant to phages isolated from the “past” versus the “future.” Phage infectivity and bacterial resistance were determined by streaking evolved bacterial colonies from time points 1, 3 and 5 across lines of phage that had previously been inoculated onto an LB agar plate. A similar method was used for bacteria isolated from transfers 3 and 5. A colony was defined as resistant if there was no inhibition of growth, otherwise it was defined as sensitive (Brockhurst, Morgan, Rainey, & Buckling, 2003; Buckling & Rainey, 2002).

### 2.8 Cost of resistance assay

To test whether bacterial resistance to phages or antibiotics was associated with a fitness cost, evolved isolates were grown in LB media in the absence of phage or antibiotic at the end of the experiment. Evolved bacteria were thawed and replicated into fresh LB using a pin replicator (~0.2 μl transfer per pin, Boekel) as described above and grown for 24 hr at 37°C. All bacterial isolates were then replicated into new 96‐well plates containing LB. Following incubation at 37°C, growth was determined as optical density at 600 nm at 24 and 48 hr postinoculation (Supporting information Figure S5). Cost of resistance was determined as the difference in bacterial maximum density or maximum growth rate of evolved relative to ancestral bacteria at 24‐hr time point.

### 2.9 Statistical analysis

Data were analysed using linear mixed models and factorial ANOVA approach where populations were set as subjects, isolation time point as a repeated factor and origin of isolation (planktonic vs. biofilm) nested under replicate populations. Bacterial density or resistance was explained by the presence of phage, presence of antibiotic and colony origin (from biofilm or planktonic phase of microcosms). The bacterial (CFU/ml and OD_600 nm_) and phage density (PFU/ml) data were log10‐transformed prior to analysis to fulfil ANOVA assumption of homogeneity of variance. When reporting results, the main effects and interactions of all factors are named before *F*‐statistics and *p*‐values.

## 3 RESULTS

### 3.1 Bacterial population density dynamics under phage and antibiotic selection

Both phage and antibiotic reduced total bacterial densities alone and in combination (treatment: *F*_3, 16_ = 62.742, *p* < 0.001; Figure 1). Antibiotic‐alone treatment was most efficient at reducing bacterial densities compared to phage‐alone treatment (antibiotic vs. phage: *df* = 16, *p* < 0.001) or phage‐antibiotic (antibiotic vs. antibiotic + phage: *df* = 16, *p* < 0.001) treatments, which were equally effective at reducing bacterial densities (phage vs. antibiotic + phage: df = 16, *p* = 0.229). However, the dynamics of density reduction caused by phage‐alone and phage‐antibiotic treatments were very different: Phage‐antibiotic treatment was initially equally effective at reducing bacterial densities as the antibiotic‐alone treatment but lost its efficiency by the third sampling (Day 9) time point, and from then on, had comparable effect on bacterial densities as the phage‐alone treatment (time × treatment: *F*_12, 16_ = 8.681, *p* < 0.001).

We found that bacterial densities were much higher in planktonic compared to biofilm populations on average (spatial dimension: *F*_1, 32_ = 335.932, *p* < 0.001). Also, the effect of phage and antibiotic was different for planktonic and biofilm populations (spatial dimension × treatment: *F*_3, 32_ = 9.17, *p* < 0.001). In planktonic populations, phage‐alone had the weakest, and phage–antibiotic intermediate and antibiotic‐alone treatment the strongest negative effect on bacterial populations (treatment: *F*_3, 16_ = 99.471, *p* < 0.001; *p* < 0.05 in all pairwise comparisons). Similar to total bacterial density analysis, phage–antibiotic treatment was initially equally effective as the antibiotic‐alone treatment but lost its efficiency by the third sampling (Day 9) time point (time × treatment: *F*_12, 16_ = 15.660, *p* < 0.001). In the case of biofilm populations, phage and antibiotic alone were equally effective at reducing bacterial densities on average, while phage–antibiotic combination was the least effective (antibiotic × phage: *F*_1, 32_ = 335.932, *p* < 0.001). All treatments became more effective at reducing the densities of biofilm populations towards the end of the experiment (time: *F*_4, 16_ = 37.116, *p* < 0.001). However, this effect was less strong in phage treatments (time × treatment: *F*_12, 16_ = 6.603, *p* < 0.001). Together, these results suggest that phage–antibiotic combination performed worse than antibiotic‐only treatment and this difference became more pronounced during the selection experiment.

### 3.2 Phage population density dynamics in the absence and presence of antibiotic selection

We found that the presence of antibiotics reduced the phage densities (antibiotic: *F*_1, 8_ = 11.090, *p* = 0.010), with combined antibiotic and phage treatment consistently having lower phage densities compared to phage‐only treatment. Phage densities increased slightly in the absence and decreased slightly in the presence of antibiotics during the experiment (phage × antibiotic: *F*_4, 32_ = 6.719 *p* < 0.001, Figure 1d). Together, these results show that antibiotics had indirect negative effect on phage densities during the selection experiment.

### 3.3 Evolution of antibiotic resistance

**Figure 2:**
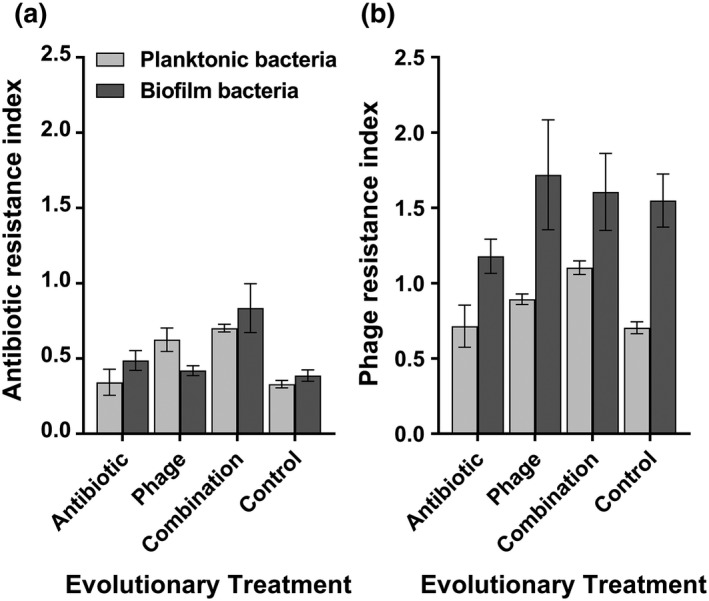
Bacterial resistance to antibiotic (a) and ancestral phage (b) measured at the end of the selection experiment. Panels (a) and (b) denote the growth of evolved bacteria in the presence of antibiotics, 3 μg/ml gentamycin and ancestral phage 14/1, respectively. Resistance indexes refer to the relative growth in the presence of antibiotic or phage compared to growth alone. Evolutionary treatment refers to different treatments during the selection experiment, and planktonic bacterial growth is shown in white and biofilm population growth in grey. Error bars show ±1 standard error of mean

Antibiotic resistance assays were conducted at the end of the selection experiment. We found that evolved bacteria had highest relative growth in the presence of antibiotics when they had previously been exposed to antibiotics and phage during the selection experiment (phage × antibiotic: *F*_1, 30_ = 5.695, *p* = 0.017; Figure 2a). Exposure to antibiotics during the selection was also shown to be linked to higher relative growth in the presence on antibiotics (antibiotic: *F*_1, 30_ = 8.398, *p* = 0.004). Prior exposure to phage selection also had a significant positive effect on bacterial growth in the presence of antibiotic (*F*_1, 30_ = 52.128, *p* < 0.001) indicative of potential cross‐resistance. A significant interaction was also found between antibiotic and phage treatments on antibiotic resistance evolution (*F*_1, 30_ = 5.695, *p* = 0.017): Although both treatments had positive effects on the evolution of antibiotic resistance alone, the effect of phage selection was relatively stronger compared to antibiotic selection, and highest levels of resistance were observed in the phage–antibiotic combination treatment. No significant difference was found between the growth of biofilm and planktonic bacterial populations in the presence of antibiotics (*p* > 0.05; Figure 2a).

### 3.4 Evolution of phage resistance

Previous exposure to a phage during the selection experiment increased bacterial growth in the presence of ancestral phage relative to the control treatment (phage: *F*_1, 30_ = 21.576, *p* < 0.001; Figure 2b). In particular, evolved bacteria isolated from the biofilms grew significantly better in the presence of a phage compared to bacteria isolated from the planktonic phase across all treatments (spatial dimension: *F*_1, 30_ = 55.134, *p* < 0.001). Antibiotic selection alone did not affect the evolution of phage resistance (antibiotic: *F*_1, 30_ = 0.315, *p* = 0.574). However, we found a significant interaction between spatial origin and phage treatment (spatial dimension × phage: *F*_1, 30_ = 9.535, *p* = 0.002), with phage selection having a larger effect on the resistance evolution of biofilm compared to planktonic populations. Together, these results suggest that phage resistance evolved to higher levels in bacterial biofilms during the selection experiment even in the absence of phage selection.

### 3.5 Bacteria–phage coevolutionary dynamics

**Figure 3:**
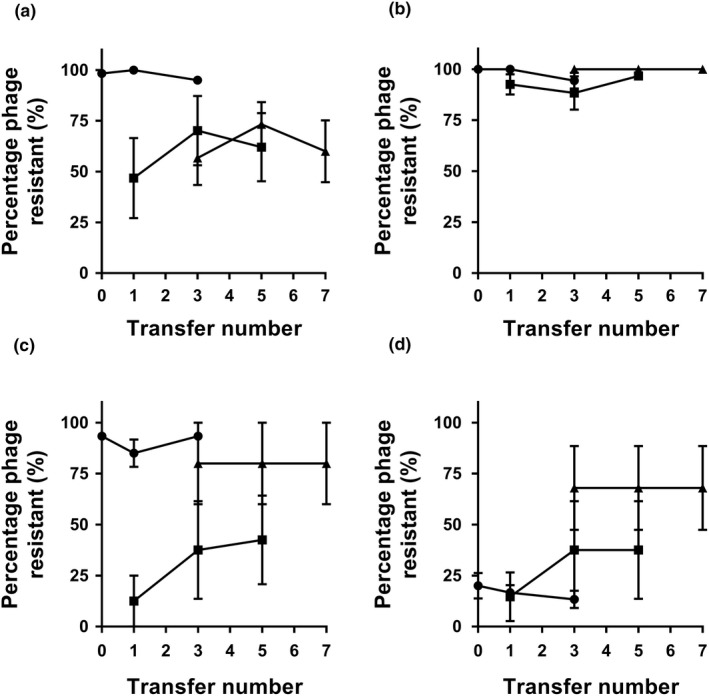
Rates of coevolution during the selection experiment for populations that evolved in planktonic (a and b) or biofilm phase (c and d) in the presence of phage (a and c) or antibiotic and phage (b and d). Each set of lines (from left to right) shows percentage of bacteria that are resistant to contemporary phage or phages isolated from two transfers in the past and in future. The slope of the line provides a measure of the rate of coevolution over each four‐transfer period. Error bars show ±1 standard error of mean

We found no clear evidence for bacteria–phage coevolutionary dynamics in any of the treatments (the effect of phage isolation time point: *F*_2, 34.6_ = 0.738, *p* = 0.485). However, distinct changes in mean phage resistance between different treatments and spatial origin were found. In contrast to endpoint measurement, phage resistance evolved to higher levels in planktonic compared to biofilm populations on average (spatial dimension: *F*_1, 48.3_ = 49.396, *p* < 0.001). Furthermore, antibiotic selection promoted the evolution of phage resistance more clearly in the planktonic bacterial populations (antibiotic × spatial dimension: *F*_1, 48.3_ = 31.981, *p* < 0.001). The rate and dynamics of phage resistance evolution also fluctuated in time depending on the treatment (time: *F*_2, 47_ = 10.403, *p* < 0.001): While phage resistance increased over time in combination treatment, more fluctuations were observed in the phage‐only treatment (Figure 3). Together, these results suggest that the study system was generally dominated by bacterial resistance evolution.

### 3.6 Quantifying the cost of resistance in the absence of phage or antibiotic

**Figure 4:**
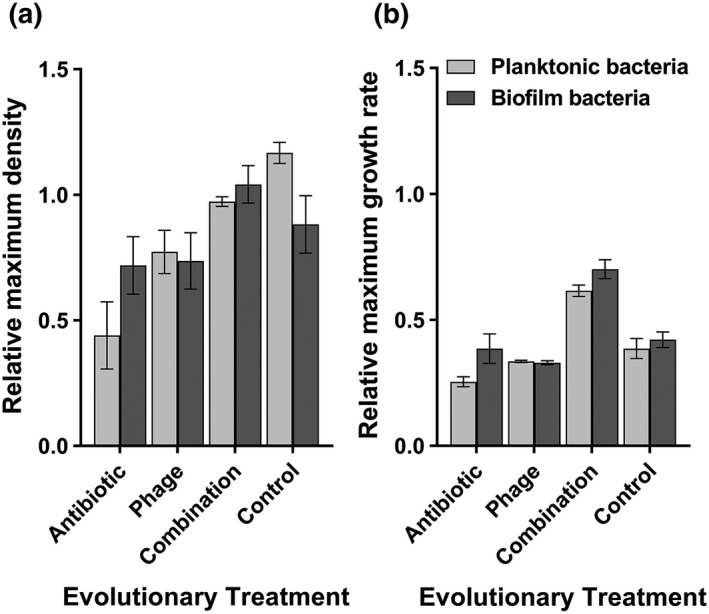
Relative cost of resistance of evolved versus ancestral bacteria after 24 hr of growth in the absence of phage or antibiotic (based on OD_600 nm_ values). (a) Maximum bacterial density of evolved bacteria relative to ancestral bacterium (dashed line). (b) Maximum growth rate of evolved bacteria relative to ancestral bacteria. In both panels, the dashed line represents the mean growth of ancestral bacterium. Evolutionary treatment refers to different treatments during the selection experiment, and planktonic bacterial growth is shown in white and biofilm population growth in grey. Error bars show ±1 standard error of mean

We found that prior evolutionary history with phage or antibiotic did not affect bacterial maximum density relative to control treatment (phage: *F*_1, 15_ = 2.884, *p* = 0.089; antibiotic: *F*_1, 15_ = 2.649, *p* = 0.104; Figure 4a). However, we found significant interaction between the two (phage × antibiotic: *F*_1, 15_ = 19.942, *p* < 0.001), which suggest that reduction in maximum density was observed in the antibiotic‐only and phage‐only but not in combination treatment. While the spatial density had nonsignificant main effect, we found a significant interaction between the antibiotic treatment and the spatial dimension (antibiotic × spatial dimension: *F*_1, 15_ = 5.024, *p* = 0.025). This suggests that bacteria treated with antibiotics suffered a larger reduction in maximum density when they were isolated from the planktonic phase of the microcosms. Cost of resistance was also measured in terms of reduction in maximum growth rate relative to ancestral bacterium (Figure 4b). Overall, all evolved populations showed clear reduction in their growth rate compared to ancestral bacterium (Figure 4b). However, this reduction was the lowest in the combination treatment (phage × antibiotic: *F*_3, 43_ = 14.43, *p* < 0.001), while spatial dimension had no significant effect on maximum growth rates. Together, these results suggest that evolution of resistance incurred relatively small cost, which was mainly observed with planktonic bacteria that had evolved in the presence of either antibiotic or the phage (maximum density), and in general, the cost of adaptation was the lowest for bacteria that were exposed to combination treatment (both maximum density and growth rate).

## 4 DISCUSSION

Here, we conducted a long‐term evolutionary study to look phage–antibiotic synergy in spatially structured environment. Even though gentamycin and the phage exerted additive effects in the short‐term preliminary experiment, we did not observe evidence of combined effects in our long‐term selection experiment. In contrast, while phage–antibiotic combination was equally good at controlling bacterial growth as antibiotic‐alone treatment in the beginning, it became less effective from Day 9 due to rapid resistance evolution. Crucially, the level of resistance evolved the highest against both antibiotics and phages in the combination treatment indicative of generalized resistance evolution. This could be explained by biofilm‐mediated resistance against both phage and antibiotic, phage‐mediated correlated selection for increased antibiotic resistance, lack of cost of adaptation and weak phage infectivity evolution. Together, these results suggest that generalized resistance mechanisms could considerably limit the efficacy of phage–antibiotic treatments in the long term.

Even though we found evidence for additive phage–antibiotic effects in our preliminary experiment, these were quickly lost during the selection experiment. Instead, phage–antibiotic combination performed worse compared to antibiotic‐alone treatment on average and was as equally effective as the antibiotic‐alone treatment for just the first 6 days. This is in contrast with previous studies reporting beneficial effects of phage–antibiotic combinations on *Escherichia coli* biofilms (Ryan, Alkawareek, Donnelly, & Gilmore, 2012) and planktonic *P. aeruginosa* cultures (Torres‐Barceló et al., 2016). However, phage–antibiotic effects are not always observed (Verma et al., 2009), which could be due to the specific phage species and class of antibiotic as PAS often requires mechanistic compatibility between the two (Chan et al., 2016; Comeau et al., 2007; Kamal & Dennis, 2015). For example, it has recently been reported that associations between phage and gentamycin resistance are predominantly positive, while associations between phage and ciprofloxacin resistance are mainly negative with natural and clinical *E. coli* isolates (Allen, Pfrunder‐Cardozo, Meinel, Egli, & Hall, 2017). In our case, potential explanation for the loss of phage–antibiotic effect is the evolution of biofilm‐mediated generalized resistance mechanism provided by the spatial heterogeneity in the microcosms.

We found that bacteria evolved more resistant to both phage and antibiotic when exposed to selection by these agents individually, and in both cases, the resistance evolved higher within biofilm compared to planktonic populations. Crucially, resistance to both selective agents evolved the highest in the phage–antibiotic combination treatment where both planktonic and biofilm populations showed equally high levels of resistance. Relatively stronger resistance evolution in combination treatment could also explain why phage population densities were consistently lower in the presence of antibiotics even though the number of host bacteria remained approximately the same between phage and phage–antibiotic treatments. The evolution of generalized resistance mechanism could be explained in various ways.

First, it is possible that selection by one selective agent led to cross‐resistance against the other agent. For example, selection by one phage or antibiotic has been shown to lead to resistance to other phages and antibiotics, respectively (Davies & Davies, 2010; Hall, De Vos, Friman, Pirnay, & Buckling, 2012). In support for this, we found that phage selection alone led to a correlated increase in antibiotic resistance, while antibiotics had contrasting effect: no effect on the phage resistance of the biofilm but slight increase in the susceptibility of the planktonic bacterial populations to the phage. Positive correlation between phage selection and antibiotic resistance is in contrast with previous studies showing an opposite effect where phage treatment reduced the emergence of antibiotic resistance (Jalasvuori, Friman, Nieminen, Bamford, & Buckling, 2011; Zhang & Buckling, 2012). One explanation for this discrepancy is that phage–antibiotic synergies could be very specific to the given phage species and type of antibiotics, whereas antibiotic selection‐mediated susceptibility to phages could have been driven by some degree of collateral sensitivity (Chan et al., 2016; Comeau et al., 2007; Kamal & Dennis, 2015).

Second, biofilms could have provided effective resistance mechanism against both phage and antibiotics (Abedon, 2017; Cochran et al., 2000; de Beer, 1997; de Beer et al., 1994; Labrie et al., 2010; Stewart & Costerton, 2001), and hence, selection for this trait could have been especially strong in the combination treatment. To explore the defensive function of biofilms in detail, we found that biofilm populations had higher levels of resistance compared to planktonic populations when exposed top selection by phage or antibiotic independently. Aminoglycoside antibiotics, including gentamycin, has been found to be less effective against biofilm than planktonic bacteria, largely due to decreased diffusion of antimicrobial agents through the biofilm matrix (Brockhurst, Buckling, & Rainey, 2006; Nouraldin et al., 2016). We found that control biofilm populations did not show clear resistance to phage or antibiotic. This suggests that biofilm growth per se did not make bacteria more resistant. Instead, biofilms likely evolved in terms of structure or other biofilm property and became more resistant to antibiotic and phage during the selection experiment. In support for this, it has been recently reported that biofilms can protect *E. coli* against phage attack via two separate mechanisms: by inhibiting phage transport into the biofilm and by coating bacterial surface and binding phage particles, thereby preventing their attachment to the cell exterior (Vidakovic et al., 2018). We also found that both planktonic and biofilm populations evolved equally high levels of resistance in the combination treatment. This suggests that in addition to biofilms, also other resistance mechanisms were important for bacterial fitness. Instead, enhanced biofilm growth could have led to more frequent sloughing of biofilm cell aggregates to the aquatic phase of the microcosms leading to increased resistance observed in planktonic population samples (Boles, Thoendel, & Singh, 2005). While these hypotheses warrant more research, our data suggest that biofilms might play very important role for the evolution of generalized resistance mechanisms in *P. aeruginosa*.

Third, while evolution of resistance is often expected to lead to fitness costs (Andersson & Hughes, 2010; Buckling, Wei, Massey, Brockhurst, & Hochberg, 2006; Forde, Thompson, Holt, & Bohannan, 2008; Ward, Perron, & MacLean, 2009), we found that the costs of adaptation were relatively small in our experiment. Moreover, costs were only observed when bacteria had been exposed to either phage or antibiotic independently, whereas no cost was observed in the combination treatment or when the bacteria were isolated from the biofilm populations in general. Similar to previous studies conducted with antibiotics (Levin, Perrot, & Walker, 2000; Perron, Hall, & Buckling, 2010), it is possible that resistant bacteria were able to acquire compensatory mutations that counteracted the costs of resistance. Alternatively, developing resistance may have incurred a small cost to begin with (Melnyk, Wong, & Kassen, 2015). In the wider context, our results suggest that evolution of generalism might not always be limited by costs of adaptation (Kassen, 2002).

One neglected aspect of phage–antibiotic combinations is the phage's ability to coevolve more infective during the treatments. In contrast to previous, relatively short‐term work with this system (Friman et al., 2016), we found no clear evidence for the coevolution between the phage and bacteria. Instead, the dynamics were mainly dictated by bacterial resistance evolution in all treatments. One explanation for this is that the spatial treatment (presence of beads) turned the coevolutionary dynamics asymmetrical by favouring highly resistant biofilm populations in the microcosms (Drenkard, 2003). If biofilms were able to completely block phage transport and access to receptors, no coevolution would be expected. However, this should have not prevented coevolution between the phage and bacteria in the planktonic populations. Lack of clear signs of coevolutionary arms race dynamics is in line with a previous study, where coevolution was observed to be dominated by bacterial resistance (Brockhurst et al., 2006). One explanation for this is that heterogeneous environments can create permanent or ephemeral spatial refuges for hosts by limiting parasite dispersal (Maynard Smith, 1974; Schrag & Mittler, 1996). Because we did not break the biofilms when refreshing the resources in the microcosms, it is likely that beads provided permanent refuges from parasites. Continuous supply and migration of resistant bacteria from biofilms would have then likely increased the proportion of resistant bacteria also in planktonic populations leading to nonobservable coevolutionary dynamics. It has also been shown that spatially structured soil environments could favour fluctuating coevolutionary dynamics and local phage–bacteria adaptation in time (Gomez & Buckling, 2011), while spatial structure could decrease bacteria–phage encounter rates leading to slower rate of coevolution (Brockhurst et al., 2006). In future, it would be interesting to test directly how the absence and presence of spatial refugees affect the host–parasite coevolutionary dynamics. In the context of phage therapy, these results suggest that spatial environment might limit the phage ability to coevolve with the bacteria at the site of infection.

In conclusion, here we show that evolution of generalized resistance mechanism can constrain the long‐term efficiency of phage–antibiotic combinations in a spatially structured environment. However, it should be noted that we used only a single phage and antibiotic combination in our experiment and it is thus somewhat unclear how far these results could be generalized. Unravelling the genetic basis of generalized resistance mechanism will be one of the next targets of research. While phage–antibiotic synergies could be driven by specific species–antibiotic combinations, it is also possible that mutation basis of adaptation plays important role. For example, in case of collateral sensitivity and cross‐resistance, it has been shown that the mutational basis of resistance to one antibiotic plays key role whether the subsequent antibiotic will have negative or neutral effect for the bacterial growth and that considerable variation can exists even between different replicate lines derived from the same selective environment (Barbosa et al., 2017). It is also likely that increasing the antibiotic concentration might change the outcome of phage–antibiotic treatments. Here, we used a sublethal concentration of gentamycin, whereas a clinically relevant concentration may have had a greater bactericidal effect without corresponding change in bacterial resistance. Moreover, even though phage–antibiotic combinations have previously been proven to be more effective than either therapy alone, these effects should be studied on evolutionary timescale to acknowledge and understand the potential complications rising due rapid resistance evolution. Our results suggest that it is important to use combinations of phage and antibiotics that are sufficiently different by necessitating mechanistic trade‐offs or substantial costs in order to prevent the rise of generalized resistance.

## DATA ARCHIVING STATEMENT

Data for this study are available at Dryad digital repository: https://doi.org/10.5061/dryad.9f77pr0

## References

[eva12653-bib-0001] Abedon, S. T. (2017). Phage “delay” towards enhancing bacterial escape from biofilms: a more comprehensive way of viewing resistance to bacteriophages. AIMS Microbiology, 3, 186–226. 10.3934/microbiol.2017.2.186 PMC6605007 31294157

[eva12653-bib-0002] Abedon, S. T. , & Thomas‐Abedon, C. (2010). Phage therapy pharmacology. Current Pharmaceutical Biotechnology, 11, 28–47. 10.2174/138920110790725410 20214606

[eva12653-bib-0003] Adams, M. H. (1959). Assay of phages by the agar layer method Bacteriophages (pp. 450–451). New York, NY: Interscience Publishers Inc.

[eva12653-bib-0004] Addy, H. S. , Askora, A. , Kawasaki, T. , Fujie, M. , & Yamada, T. (2012). Loss of virulence of the phytopathogen ralstonia solanacearum through infection by φRSM filamentous phages. Phytopathology, 102, 469–477. 10.1094/PHYTO-11-11-0319-R 22352303

[eva12653-bib-0005] Allen, R. C. , Pfrunder‐Cardozo, K. R. , Meinel, D. , Egli, A. , & Hall, A. R. (2017). Associations among antibiotic and phage resistance phenotypes in natural and clinical *Escherichia coli* isolates. mBio, 8, e01341–17. 29089428 10.1128/mBio.01341-17 PMC5666156

[eva12653-bib-0006] Andersson, D. I. , & Hughes, D. (2010). Antibiotic resistance and its cost: Is it possible to reverse resistance? Nature Reviews Microbiology, 8, 260–271. 10.1038/nrmicro2319 20208551

[eva12653-bib-0007] Barbosa, C. , Trebosc, V. , Kemmer, C. , Rosenstiel, P. , Beardmore, R. , Schulenburg, H. , & Jansen, G. (2017). Alternative evolutionary paths to bacterial antibiotic resistance cause distinct collateral effects. Molecular Biology and Evolution, 34, 2229–2244. 10.1093/molbev/msx158 28541480 PMC5850482

[eva12653-bib-0008] de Beer, D. (1997). Measurement of local diffusion coefficients in biofilms by micro‐injection and confocal microscopy. Biotechnology and Bioengineering, 53, 151–158. 10.1002/(ISSN)1097-0290 18633959

[eva12653-bib-0009] de Beer, D. , Stoodley, P. , Roe, F. , & Lewandowski, Z. (1994). Effects of biofilm structures on oxygen distribution and mass transport. Biotechnology and Bioengineering, 43, 1131–1138. 10.1002/(ISSN)1097-0290 18615526

[eva12653-bib-0010] Betts, A. , Vasse, M. , Kaltz, O. , & Hochberg, M. E. (2013). Back to the future: evolving bacteriophages to increase their effectiveness against the pathogen *Pseudomonas aeruginosa* PAO1. Evolutionary Applications, 6, 1054–1063. 24187587 10.1111/eva.12085 PMC3804238

[eva12653-bib-0011] Boles, B. R. , Thoendel, M. , & Singh, P. K. (2005). Rhamnolipids mediate detachment of *Pseudomonas aeruginosa* from biofilms. Molecular Microbiology, 57, 1210–1223. 10.1111/j.1365-2958.2005.04743.x 16101996

[eva12653-bib-0012] Brockhurst, M. A. , Buckling, A. , & Rainey, P. B. (2006). Spatial heterogeneity and the stability of host‐parasite coexistence. Journal of Evolutionary Biology, 19, 374–379. 10.1111/j.1420-9101.2005.01026.x 16599913

[eva12653-bib-0013] Brockhurst, M. A. , Morgan, A. D. , Rainey, P. B. , & Buckling, A. (2003). Population mixing accelerates coevolution. Ecology Letters, 6, 975–979. 10.1046/j.1461-0248.2003.00531.x

[eva12653-bib-0014] Buckling, A. , & Rainey, P. B. (2002). Antagonistic coevolution between a bacterium and a bacteriophage. Proceedings of the Royal Society B: Biological Sciences, 269, 931–936. 10.1098/rspb.2001.1945 12028776 PMC1690980

[eva12653-bib-0015] Buckling, A. , Wei, Y. , Massey, R. C. , Brockhurst, M. A. , & Hochberg, M. E. (2006). Antagonistic coevolution with parasites increases the cost of host deleterious mutations. Proceedings of the Royal Society B: Biological Sciences, 273, 45–49. 10.1098/rspb.2005.3279 16519233 PMC1560003

[eva12653-bib-0016] Carlton, R. M. (1999). Phage therapy: Past history and future prospects. Archivum Immunologiae et Therapiae Experimentalis, 47, 267–274. 10604231

[eva12653-bib-0017] Chan, B. K. , Sistrom, M. , Wertz, J. E. , Kortright, K. E. , Narayan, D. , & Turner, P. E. (2016). Phage selection restores antibiotic sensitivity in MDR *Pseudomonas aeruginosa* . Scientific Reports, 6, 26717 10.1038/srep26717 27225966 PMC4880932

[eva12653-bib-0018] Chaturongakul, S. , & Ounjai, P. (2014). Phage‐host interplay: Examples from tailed phages and Gram‐negative bacterial pathogens. Frontiers in Microbiology, 5, 1–8. 25191318 10.3389/fmicb.2014.00442 PMC4138488

[eva12653-bib-0019] Chaudhry, W. N. , Concepción‐Acevedo, J. , Park, T. , Andleeb, S. , Bull, J. J. , & Levin, B. R. (2017). Synergy and order effects of antibiotics and phages in killing *pseudomonas aeruginosa* biofilms. PLoS ONE, 12, 1–16. 10.1371/journal.pone.0168615 PMC5226664 28076361

[eva12653-bib-0020] Cochran, W. L. , McFeters, G. A. , & Stewart, P. S. (2000). Reduced susceptibility of thin *Pseudomonas aeruginosa* biofilms to hydrogen peroxide and monochloramine. Journal of Applied Microbiology, 88, 22–30. 10735239 10.1046/j.1365-2672.2000.00825.x

[eva12653-bib-0021] Comeau, A. M. , Tétart, F. , Trojet, S. N. , Prère, M. F. , & Krisch, H. M. (2007). Phage‐antibiotic synergy (PAS): β‐lactam and quinolone antibiotics stimulate virulent phage growth. PLoS ONE, 2, e799 10.1371/journal.pone.0000799 17726529 PMC1949050

[eva12653-bib-0022] Costerton, J. W. , Stewart, P. S. , & Greenberg, E. P. (1999). Bacterial biofilms: A common cause of persistent infections. Science, 284, 1318–1322. 10.1126/science.284.5418.1318 10334980

[eva12653-bib-0023] Davies, J. C. (2002). *Pseudomonas aeruginosa* in cystic fibrosis: Pathogenesis and persistence. Paediatric Respiratory Reviews, 3, 128–134. 10.1016/S1526-0550(02)00003-3 12297059

[eva12653-bib-0024] Davies, J. , & Davies, D. (2010). Origins and evolution of antibiotic resistance. Microbiology and Molecular Biology Reviews, 74, 417–433. 10.1128/MMBR.00016-10 20805405 PMC2937522

[eva12653-bib-0025] Donlan, R. M. , & Costerton, J. W. (2002). Biofilms: Survival mechanisms of clinically relevant microorganisms. Clinical Microbiology Reviews, 15, 167–19. 10.1128/CMR.15.2.167-193.2002 11932229 PMC118068

[eva12653-bib-0026] Doolittle, M. M. , Cooney, J. J. , & Caldwell, D. E. (1996). Tracing the interaction of bacteriophage with bacterial biofilms using fluorescent and chromogenic probes. Journal of Industrial Microbiology, 16, 331–341. 10.1007/BF01570111 8987490

[eva12653-bib-0027] Drenkard, E. (2003). Antimicrobial resistance of *Pseudomonas aeruginosa* biofilms. Microbes and Infection, 5, 1213–1219. 10.1016/j.micinf.2003.08.009 14623017

[eva12653-bib-0028] Forde, S. E. , Thompson, J. N. , Holt, R. D. , & Bohannan, B. J. M. (2008). Coevolution drives temporal changes in fitness and diversity across environments in a bacteria‐bacteriophage interaction. Evolution, 62, 1830–1839. 18452575 10.1111/j.1558-5646.2008.00411.x

[eva12653-bib-0029] Friman, V.‐P. , Soanes‐Brown, D. , Sierocinski, P. , Molin, S. , Johansen, H. K. , Merabishvili, M. , … Buckling, A. (2016). Pre‐adapting parasitic phages to a pathogen leads to increased pathogen clearance and lowered resistance evolution with *Pseudomonas aeruginosa* cystic fibrosis bacterial isolates. Journal of Evolutionary Biology, 29, 188–198. 10.1111/jeb.12774 26476097

[eva12653-bib-0030] Gomez, P. , & Buckling, A. (2011). Bacteria‐phage antagonistic coevolution in soil. Science, 332, 106–109. 10.1126/science.1198767 21454789

[eva12653-bib-0031] Hagens, S. , Habel, A. , & Blasi, U. (2006). Augmentation of the antimicrobial efficacy of antibiotics by filamentous phage. Microbial Drug Resistance, 12, 164–168. 10.1089/mdr.2006.12.164 17002542

[eva12653-bib-0032] Hall, A. R. , De Vos, D. , Friman, V.‐P. , Pirnay, J. P. , & Buckling, A. (2012). Effects of sequential and simultaneous applications of bacteriophages on populations of *Pseudomonas aeruginosa* in vitro and in wax moth larvae. Applied and Environmental Microbiology, 78, 5646–5652. 10.1128/AEM.00757-12 22660719 PMC3406105

[eva12653-bib-0033] Hanlon, G. W. , Denyer, S. P. , Olliff, C. J. , & Ibrahim, L. J. (2001). Reduction in exopolysaccharide viscosity as an aid to bacteriophage penetration through *Pseudomonas aeruginosa* biofilms. Applied and Environmental Microbiology, 67, 2746–2753. 10.1128/AEM.67.6.2746-2753.2001 11375190 PMC92934

[eva12653-bib-0034] Huff, W. E. , Huff, G. R. , Rath, N. C. , Balog, J. M. , & Donoghue, A. M. (2004). Therapeutic efficacy of bacteriophage and Baytril (enrofloxacin) individually and in combination to treat colibacillosis in broilers. Poultry Science, 83, 1944–1947. 10.1093/ps/83.12.1944 15615004

[eva12653-bib-0035] Hughes, K. A. , Sutherland, I. W. , Jones, M. V. , & Rutherford, D. (1998). Biofilm susceptibility to bacteriophage attack: The role of phage‐borne polysaccharide depolymerase. Microbiology, 144, 3039–3047. 10.1099/00221287-144-11-3039 9846739

[eva12653-bib-0036] Imamovic, L. , & Sommer, M. O. A. (2013). Use of collateral sensitivity networks to design drug cycling protocols that avoid resistance development. Science Translational Medicine, 5, 204ra132. 10.1126/scitranslmed.3006609 24068739

[eva12653-bib-0037] Jalasvuori, M. , Friman, V.‐P. , Nieminen, A. , Bamford, J. K. H. , & Buckling, A. (2011). Bacteriophage selection against a plasmid‐encoded sex apparatus leads to the loss of antibiotic‐resistance plasmids. Biology Letters, 7, 902–905. 10.1098/rsbl.2011.0384 21632619 PMC3210665

[eva12653-bib-0038] Kamal, F. , & Dennis, J. J. (2015). Burkholderia cepacia complex phage‐antibiotic synergy (PAS): Antibiotics stimulate lytic phage activity. Applied and Environmental Microbiology, 81, 1132–1138. 10.1128/AEM.02850-14 25452284 PMC4292504

[eva12653-bib-0039] Kassen, R. (2002). The experimental evolution of specialists, generalists, and the maintenance of diversity. Journal of Evolutionary Biology, 15, 173–190. 10.1046/j.1420-9101.2002.00377.x

[eva12653-bib-0040] Kutter, E. M. , De Vos, D. , Gvasalia, G. , Alavidze, Z. , Gogokhia, L. , Kuhl, S. J. , & Abedon, S. T. (2010). Phage therapy in clinical practice: Treatment of human infections. Current Pharmaceutical Biotechnology, 11, 69–86. 10.2174/138920110790725401 20214609

[eva12653-bib-0041] Labrie, S. J. , Samson, J. E. , & Moineau, S. (2010). Bacteriophage resistance mechanisms. Nature Reviews Microbiology, 8, 317–327. 10.1038/nrmicro2315 20348932

[eva12653-bib-0042] Levin, B. R. , Perrot, V. , & Walker, N. (2000). Compensatory mutations, antibiotic resistance and the population genetics of adaptive evolution in bacteria. Genetics, 154, 985–997. 10757748 10.1093/genetics/154.3.985 PMC1460977

[eva12653-bib-0043] Lister, P. D. , Wolter, D. J. , & Hanson, N. D. (2009). Antibacterial‐resistant *Pseudomonas aeruginosa*: Clinical impact and complex regulation of chromosomally encoded resistance mechanisms. Clinical Microbiology Reviews, 22, 582–610. 10.1128/CMR.00040-09 19822890 PMC2772362

[eva12653-bib-0044] Maynard Smith, J. (1974). Models in Ecology. Cambridge: Cambridge University Press.

[eva12653-bib-0045] Melnyk, A. H. , Wong, A. , & Kassen, R. (2015). The fitness costs of antibiotic resistance mutations. Evolutionary Applications, 8, 273–283. 10.1111/eva.12196 25861385 PMC4380921

[eva12653-bib-0046] Merabishvili, M. , Verhelst, R. , Glonti, T. , Chanishvili, N. , Krylov, V. , Cuvelier, C. , … Vaneechoutte, M. (2007). Digitized fluorescent RFLP analysis (fRFLP) as a universal method for comparing genomes of culturable dsDNA viruses: Application to bacteriophages. Research in Microbiology, 158, 572–581. 10.1016/j.resmic.2007.06.002 17719750

[eva12653-bib-0047] Mumford, R. , & Friman, V.‐P. (2017). Bacterial competition and quorum‐sensing signalling shape the eco‐evolutionary outcomes of model in vitro phage therapy. Evolutionary Applications, 10, 161–169. 10.1111/eva.12435 28127392 PMC5253424

[eva12653-bib-0048] Nee, S. (1989). Antagonistic co‐evolution and the evolution of genotypic randomization. Journal of Theoretical Biology, 140, 499–518. 10.1016/S0022-5193(89)80111-0 2615403

[eva12653-bib-0049] Nouraldin, A. A. M. , Baddour, M. M. , Harfoush, R. A. H. , & Essa, S. A. M. (2016). Bacteriophage‐antibiotic synergism to control planktonic and biofilm producing clinical isolates of *Pseudomonas aeruginosa* . Alexandria Journal of Medicine, 52, 99–105. 10.1016/j.ajme.2015.05.002

[eva12653-bib-0050] O'Neill, J. (2014). Antimicrobial Resistance: Tackling a crisis for the health and wealth of nations. Review on Antimicrobial Resistance.

[eva12653-bib-0051] Percival, S. L. , Suleman, L. , & Donelli, G. (2015). Healthcare‐Associated infections, medical devices and biofilms: Risk, tolerance and control. Journal of Medical Microbiology, 64, 323–334. 10.1099/jmm.0.000032 25670813

[eva12653-bib-0052] Perron, G. G. , Hall, A. R. , & Buckling, A. (2010). Hypermutability and compensatory adaptation in antibiotic‐resistant bacteria. The American Naturalist, 176, 303–311. 10.1086/655217 20624092

[eva12653-bib-0053] Popat, R. , Crusz, S. A. , Messina, M. , Williams, P. , West, S. A. , & Diggle, S. P. (2012). Quorum‐sensing and cheating in bacterial biofilms. Proceedings of the Royal Society B: Biological Sciences, 279, 4765–4771. 10.1098/rspb.2012.1976 23034707 PMC3497100

[eva12653-bib-0054] Ryan, E. M. , Alkawareek, M. Y. , Donnelly, R. F. , & Gilmore, B. F. (2012). Synergistic phage‐antibiotic combinations for the control of Escherichia coli biofilms in vitro. FEMS Immunology and Medical Microbiology, 65, 395–398. 10.1111/j.1574-695X.2012.00977.x 22524448

[eva12653-bib-0055] Scanlan, P. D. , Buckling, A. , & Hall, A. R. (2015). Experimental evolution and bacterial resistance: (co)evolutionary costs and trade‐offs as opportunities in phage therapy research. Bacteriophage, 5, e1050153 10.1080/21597081.2015.1050153 26459626 PMC4588173

[eva12653-bib-0056] Schrag, S. J. , & Mittler, J. E. (1996). Host‐parasite coexistence: The role of spatial refuges in stabilizing bacteria‐phage interactions. The American Naturalist, 148, 348–376. 10.1086/285929

[eva12653-bib-0057] Skurnik, M. , Pajunen, M. , & Kiljunen, S. (2007). Biotechnological challenges of phage therapy. Biotechnology Letters, 29, 995–1003. 10.1007/s10529-007-9346-1 17364214

[eva12653-bib-0058] Stewart, P. S. , & Costerton, J. W. (2001). Antibiotic resistance of bacteria in biofilms. The Lancet, 358, 135–138. 10.1016/S0140-6736(01)05321-1 11463434

[eva12653-bib-0059] Stover, C. K. , Pham, X. Q. , Erwin, A. L. , Mizoguchi, S. D. , Warrener, P. , Hickey, M. J. , … Olson, M. V. (2000). Complete genome sequence of *Pseudomonas aeruginosa* PAO1, an opportunistic pathogen. Nature, 406, 959–964. 10.1038/35023079 10984043

[eva12653-bib-0060] Torres‐Barceló, C. , Franzon, B. , Vasse, M. , & Hochberg, M. E. (2016). Long‐term effects of single and combined introductions of antibiotics and bacteriophages on populations of *Pseudomonas aeruginosa* . Evolutionary Applications, 9, 583–595. 10.1111/eva.12364 27099623 PMC4831460

[eva12653-bib-0061] Torres‐Barceló, C. , & Hochberg, M. E. (2016). Evolutionary rationale for phages as complements of antibiotics. Trends in Microbiology, 24, 249–256. 10.1016/j.tim.2015.12.011 26786863

[eva12653-bib-0062] Trautner, B. W. , & Darouiche, R. O. (2010). Role of biofilm in catheter‐associated urinary tract infection. American Journal of Infection Control, 32, 177–183. 10.1016/j.ajic.2003.08.005 PMC2963581 15153930

[eva12653-bib-0063] Traverse, C. C. , Mayo‐Smith, L. M. , Poltak, S. R. , & Cooper, V. S. (2013). Tangled bank of experimentally evolved Burkholderia biofilms reflects selection during chronic infections. Proceedings of the National Academy of Sciences, 110, E250–E259. 10.1073/pnas.1207025110 PMC3549113 23271804

[eva12653-bib-0064] Tuomanen, E. , Cozens, R. , Tosch, W. , Zak, O. , & Tomasz, A. (1986). The rate of killing of *Escherichia coli* by ‐Lactam antibiotics is strictly proportional to the rate of bacterial growth. Microbiology, 132, 1297–1304. 10.1099/00221287-132-5-1297 3534137

[eva12653-bib-0065] Verma, V. , Harjai, K. , & Chhibber, S. (2009). Restricting ciprofloxacin‐induced resistant variant formation in biofilm of Klebsiella pneumoniae B5055 by complementary bacteriophage treatment. Journal of Antimicrobial Chemotherapy, 64, 1212–1218. 10.1093/jac/dkp360 19808232

[eva12653-bib-0066] Vidakovic, L. , Singh, P. K. , Hartmann, R. , Nadell, C. D. , & Drescher, K. (2018). Dynamic biofilm architecture confers individual and collective mechanisms of viral protection. Nature Microbiology, 3, 26–31. 10.1038/s41564-017-0050-1 PMC5739289 29085075

[eva12653-bib-0067] Wang, X. , Wei, Z. , Li, M. , Wang, X. , Shan, A. , Mei, X. , … Friman, V.‐P. (2017). Parasites and competitors suppress bacterial pathogen synergistically due to evolutionary trade‐offs. Evolution, 71, 733–746. 10.1111/evo.13143 27925169 PMC5347860

[eva12653-bib-0068] Ward, H. , Perron, G. G. , & MacLean, R. C. (2009). The cost of multiple drug resistance in *Pseudomonas aeruginosa* . Journal of Evolutionary Biology, 22, 997–1003. 10.1111/j.1420-9101.2009.01712.x 19298493

[eva12653-bib-0069] Zhang, Q. G. , & Buckling, A. (2012). Phages limit the evolution of bacterial antibiotic resistance in experimental microcosms. Evolutionary Applications, 5, 575–582. 10.1111/j.1752-4571.2011.00236.x 23028398 PMC3461140

