# Supplementary material for: Rapid evolution of generalized resistance mechanisms can constrain the efficacy of phage–antibiotic treatments
Source: Evol Appl. 2018 Jun 21;11(9):1630–41. doi: 10.1111/eva.12653 (PMC6183449; doi:10.1111/eva.12653)
Supplement: Supplementary file 1 [file EVA-11-1630-s001.docx]

# **SUPPLEMENTARY MATERIALS**

**Experimental design**

**
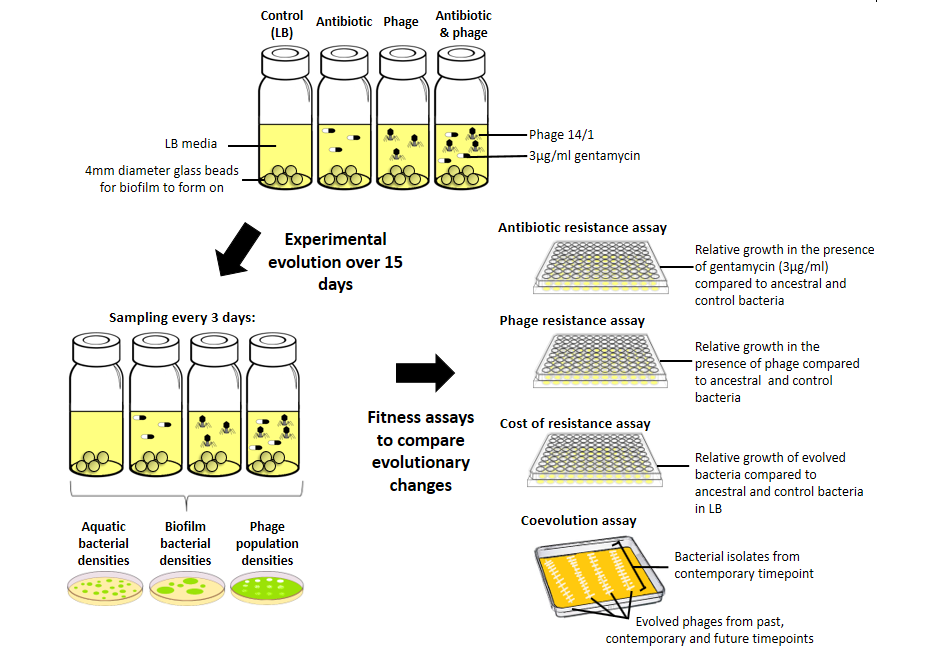
**

**Supplementary figure 1.** Experimental setup. Microcosms containing 25 glass beads and cultures of Pseudomonas aeruginosa PAO1 were evolved over 15 days in the presence or absence of gentamycin and bacteriophage 14/1. Microcosms were sampled every 3 days to quantify bacterial (planktonic and biofilm populations) and phage population densities and isolates from each time point were cryopreserved at -80°C. After the selection experiment, evolved bacterial clones and phage populations were isolated for fitness assays using the last time point. To measure evolutionary changes, evolved bacterial isolates were grown in the presence of gentamycin (to quantify antibiotic resistance), ancestral phage (to quantify phage resistance), and LB (to quantify the cost of resistance). The phage resistance of evolved bacterial isolates were also tested against phages isolated from the past, contemporary and future time points to quantify coevolutionary changes.

**Determining initial effects of phage, antibiotic and phage and antibiotic combinations on *P. aeruginosa* PAO1 growth**

Minimum inhibitory concentration (MIC) of gentamycin was determined experimentally to quantify whether the presence of phage 14/1 would reduce the bacterial growth beyond the effect of antibiotic only. Cultures of phage-sensitive PAO1 were grown for 24 hours at 37^o^C with shaking at 200rpm. Approximately 10^7^ cells were inoculated into 200 µl of LB containing increasing gentamycin concentrations (0µg mL^-1^ - 24µg mL^-1^) in a 96-well plate. Six replicates were made per treatment. Cultures were incubated at 37^o^C and growth was measured at 24 hours using optical density readings at 600nm on a microplate reader.


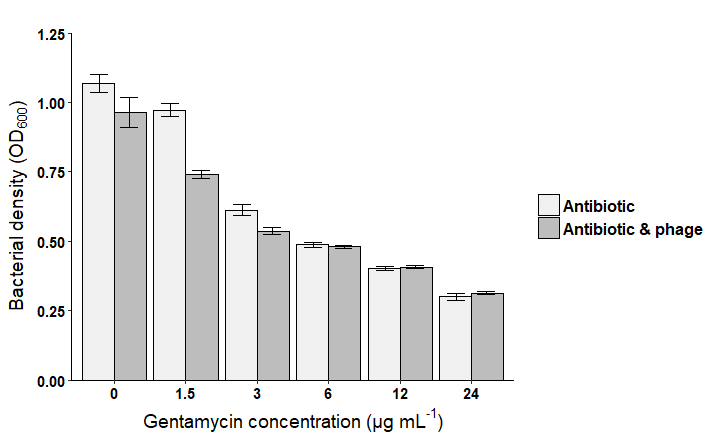


***Supplementary figure 2.*** *The effect of antibiotic-only treatment (white) and antibiotic and phage combination treatment (grey) on bacterial density when grown for 24 hours. Error bars show ±1 standard error of mean.*

Minimum inhibitory concentration for PAO1 was defined as the lowest concentration of gentamycin resulting in inhibition of bacterial growth and was found to be 24 µg mL^-1^ gentamycin (Supplementary Fig. 2). MIC was also calculated in the presence of phages to determine whether phages affected the efficacy of antibiotic treatment. Phage were found to significantly reduce density beyond that of antibiotic when the antibiotic concentration was below 6 µg mL^-1^. A sub-lethal concentration of 3 µg mL^-1^ gentamycin was therefore chosen for the evolutionary experiment to act as a selective pressure without killing all bacteria outright, even in combination with 14/1.

**The effect of temperature on the efficacy of gentamycin antibiotic when stored at 4°C or 37°C**

**
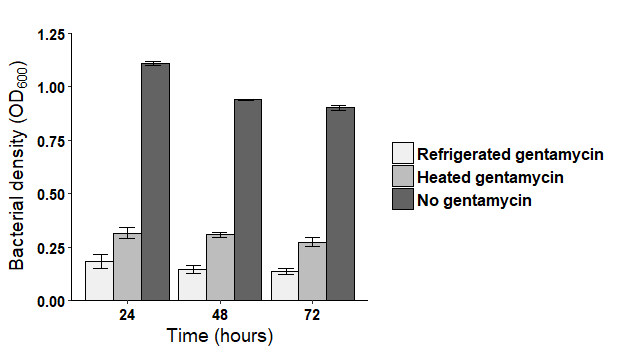
**

***Supplementary figure 3.*** *The effect of gentamycin on P. aeruginosa PAO1 densities when mixed with LB and stored at 4°C or 37°C (heated). Refrigerated gentamycin (white) refers to 3μg mL^-1^ gentamycin in LB stored at 4^o^C for 72 hours. Heated gentamycin (light grey) refers to 3μg/mL gentamycin in LB stored at 37^o^C for 72 hours, equivalent to antibiotic treatment used in the central evolutionary experiment. No gentamycin (dark grey) refers to PAO1 grown in LB alone. Error bars show ±1 standard error of mean.*

**Antibiotic and phage resistance growth data used to count the resistance indexes**


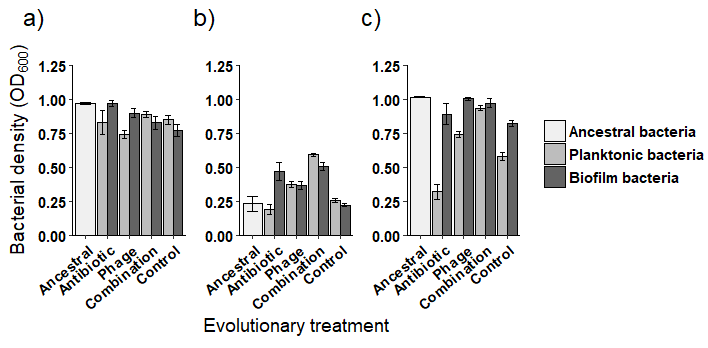


***Supplementary figure 4.*** *The* *mean densities (OD_600_) of evolved bacteria 24 hours after inoculation when grown in (a) LB only, (b) LB containing 3 µg mL^-1^ gentamycin or (c) LB containing ancestral phage 14/1.* *Mean ancestral bacterial density is shown in white, planktonic bacterial densities light grey and biofilm bacterial densities in dark grey. Evolutionary treatment refers to treatments during the selection experiment and error bars show ±1 standard error of mean.*

**Cost of resistance extended figures**

**
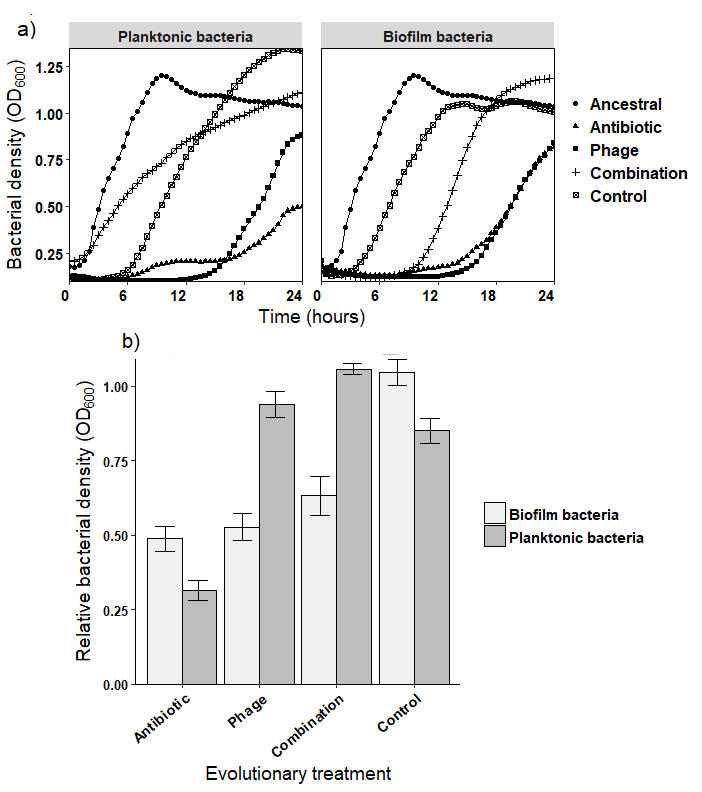
**

***Supplementary figure 5.*** *Cost of resistance measured in the absence of phage or antibiotic. (a) Growth curves of evolved bacteria and ancestral bacteria in LB over 24 hours. (b) Proportional density of evolved bacteria relative to ancestral bacteria after 48 hours of growth in LB. Evolutionary treatment refers to conditions during the selection experiment and planktonic bacterial growth is shown in white and biofilm population growth in grey. Error bars show ±1 standard error of mean.*
